# Supplementary material for: Pro-Resolving Lipid Mediator Resolvin E1 Mitigates the Progress of Diethylnitrosamine-Induced Liver Fibrosis in Sprague-Dawley Rats by Attenuating Fibrogenesis and Restricting Proliferation
Source: Int J Mol Sci. 2020 Nov 22;21(22):8827. doi: 10.3390/ijms21228827 (PMC7700193; doi:10.3390/ijms21228827)
Supplement: Supplementary file 1 [file ijms-21-08827-s001.pdf]

Supplementary

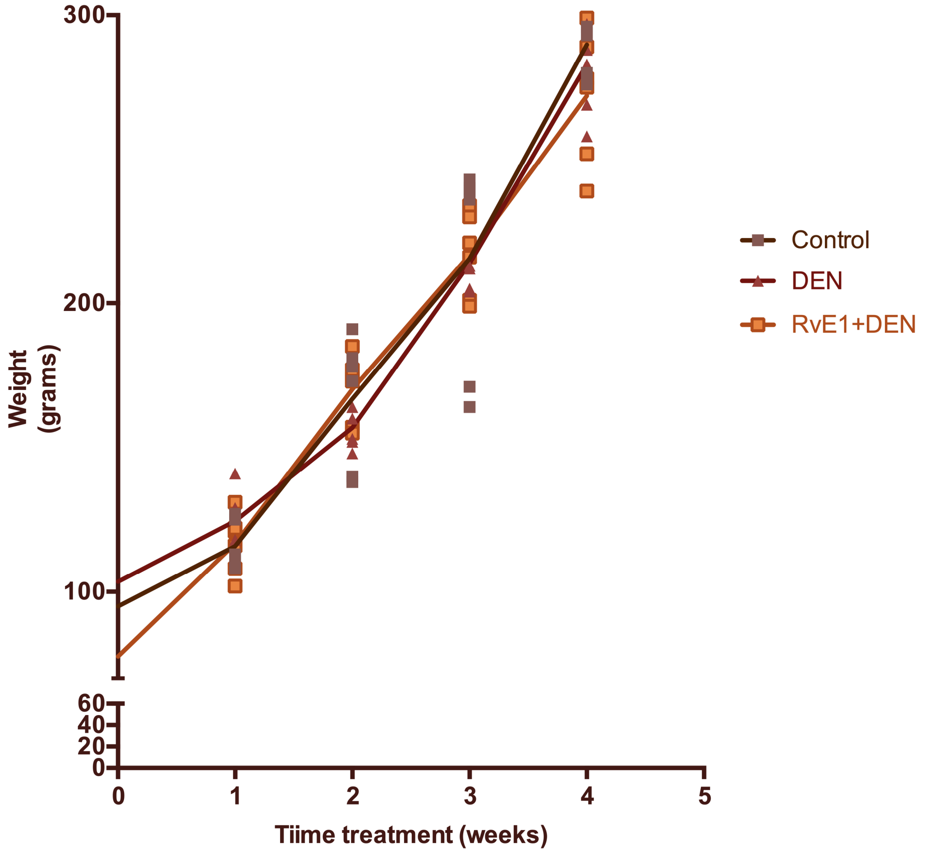


Figure 1: Growth chart

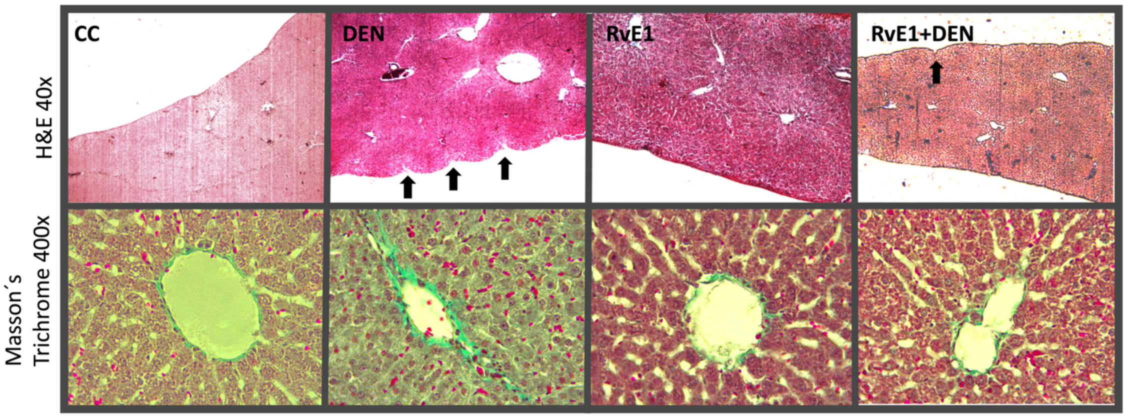


Figure 2:  Upper image correspond to H&E 40X, lower image Masson´s trichome 400x central vein detail and ECM deposit.
